# Supplementary material for: Over-Expressed GATA-1S, the Short Isoform of the Hematopoietic Transcriptional Factor GATA-1, Inhibits Ferroptosis in K562 Myeloid Leukemia Cells by Preventing Lipid Peroxidation
Source: Antioxidants (Basel). 2023 Feb 21;12(3):537. doi: 10.3390/antiox12030537 (PMC10045147; doi:10.3390/antiox12030537)
Supplement: Supplementary file 1 [file antioxidants-12-00537-s001.zip › antioxidants-2111989-supplementary.pdf]

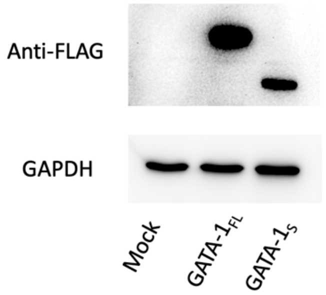

Figure S1: Western blot analysis of the expression levels of GATA-1 isoforms in total protein lysates from K562 cells transiently transfected with either FLAG-tagged GATA-1FL (48 kD) or GATA-1S (38 kD) expression vectors or empty vector (mock control).

Table S1. List of fatty acids (and cholesterol) detected by GC-MS. Name, chain length, similarity, retention time (R.T.), and retention index (R.I.) are indicated for each compound.

#	*Name*	*Chain Length*	*Similarity*	*R.T. (s)*	*RI*	*RI NIST*	*Delta RI*
1	Octanoic acid, methyl ester	C8:0	897	359.006	1140	**1126**	-14
2	Nonanoic acid, methyl ester	C9:0	870	405.744	1219	**1225**	6
3	Dodecanoic acid, methyl ester	C12:0	876	623.404	1517	**1526**	9
4	Methyl tetradecanoate	C14:0	948	838.181	1723	**1725**	2
5	Pentadecanoic acid, methyl ester	C15:0	910	952.068	1825	**1820**	-5
6	7-Hexadecenoic acid, methyl ester, (Z)-	C16:1ω9	925	1037.23	1901	**1900**	-1
7	9-Hexadecenoic acid, methyl ester, (Z)-	C16:1ω7	815	1060.22	1922	**1898**	-24
8	Hexadecanoic acid, methyl ester	C16:0	953	1067.38	1928	**1926**	-2
9	cis-10-Heptadecenoic acid, methyl ester	C17:1ω7	904	1151.08	2015	**2003**	-12
10	Heptadecanoic acid, methyl ester	C17:0	898	1177.07	2027	**2028**	1
11	9,12-Octadecadienoic acid (Z,Z)-, methyl ester	C18:2ω6	919	1251.12	2097	**2098**	1
12	9-Octadecenoic acid (Z)-, methyl ester	C18:1ω9(Z)	950	1259.82	2105	**2091**	-14
13	9-Octadecenoic acid (E)-, methyl ester	C18:1ω9(E)	949	1264.63	2109	**2105**	-4
14	Methyl stearate	C18:0	947	1286.79	2130	**2128**	-2
15	5,8,11,14-Eicosatetraenoic acid, methyl ester, (all-Z)-	C20:4	907	1421.83	2261	**2274**	13
16	5,8,11,14,17-Eicosapentaenoic acid, methyl ester, (all-Z)-	C20:5ω3	848	1428.57	2269	**2282**	-13
17	Eicosanoic acid, methyl ester	C20:0	938	1490.08	2330	**2329**	-1
18	4,7,10,13,16,19-Docosahexaenoic acid, methyl ester, (all-Z)-	C22:6	922	1609.69	2455	**2471**	16
19	cis-7,10,13,16-Docosatetraenoic acid, methyl ester	C22:4ω6	842	1615.91	2462	**2481**	19
20	Docosapentaenoic Acid methyl ester	C22:5ω3	897	1622.99	2469	**2515**	46
21	13-Docosenoic acid, methyl ester, (Z)-	C22:1ω9	831	1651.96	2501	**2499**	-2
22	Tetracosanoic acid, methyl ester	C25:0	902	1856.69	2733	**2728**	-5
23	Cholesterol	-	906	2178.19	3114	**3098**	-16

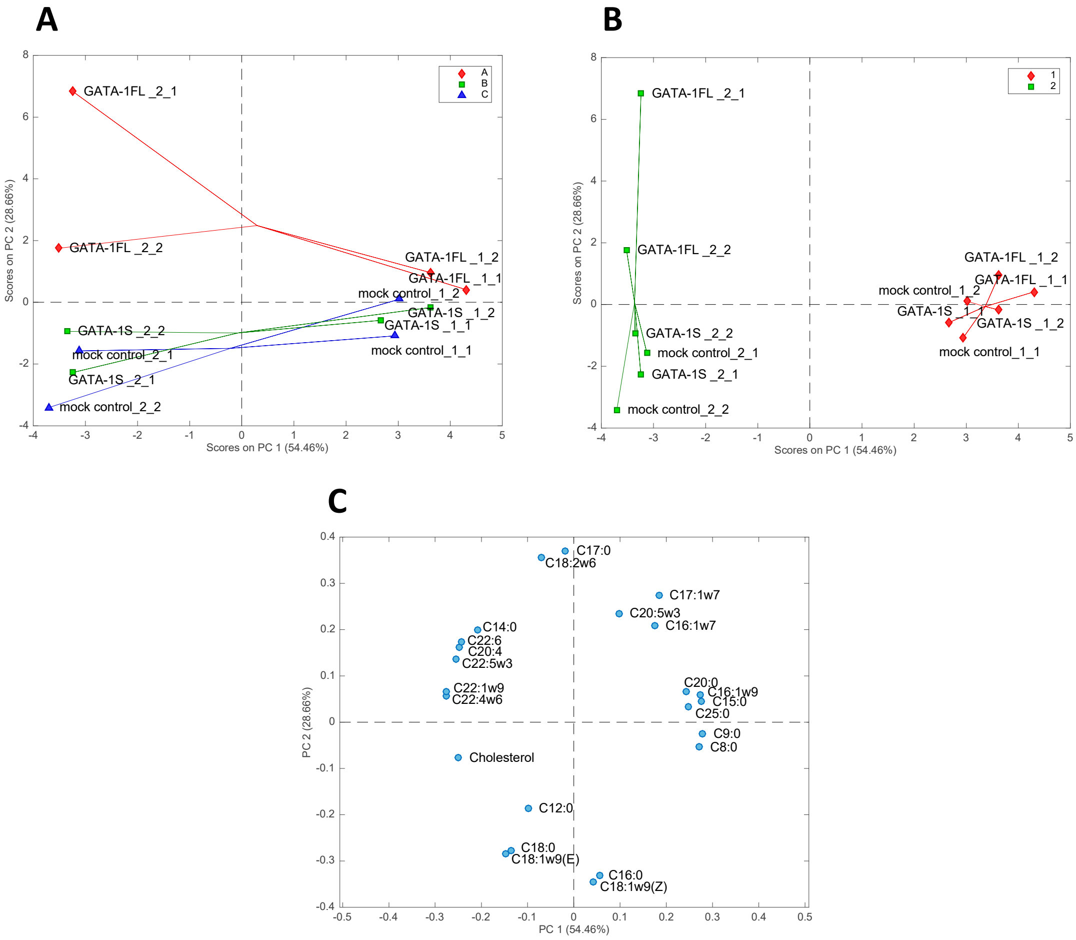

Figure S2. Scores (top) and loadings (bottom) plots derived from the PCA model, including the three studied groups, obtained by the deconvolution of the GC-MS data. The scores plot have been colored according to (A) transfection vector and (B) batch (biological replicates).

Table S2. ASCA-based decomposition of the variation according to the two main effects, transfection vector and batch and the two-factor interaction effect by considering all the investigated samples (n = 12). PCs stands for Principal Components.

Factor	PCs	Variance (%)	*P*-value
Transfection vector	2	19.21	0.0075
Batch	1	53.71	0.0005
(Transf. vector) x (Batch)	2	13.25	0.0190
Mean		0.00	
Residuals		13.82	

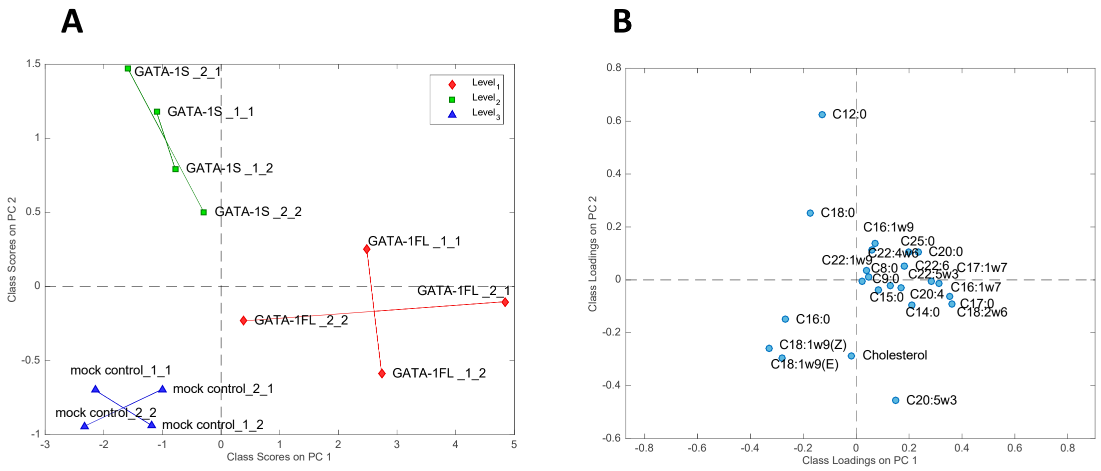

Figure S3. Scores (A) and loadings (B) plots derived from the $X_{transf.vector}$ obtained by the ASCA.

Table S3. ASCA-based decomposition of the variation according to the two main effects, transfection vector and batch and the two-factor interaction effect by considering only two types of transfection vector (n = 8). PCs stands for Principal Components.

Factor	***PCs***	**Variance (%)**	***P-value***
Transfection vector	1	16.74	0.0400
Batch	1	56.54	0.0005
(Transf. vector) x (Batch)	1	9.53	0.1210
Mean		0.00	
Residuals		17.18	
